# Influence of Diet and *Postmortem* Ageing on Oxidative Stability of Lipids, Myoglobin and Myofibrillar Proteins and Quality Attributes of *Gluteus Medius* Muscle in Goats

**DOI:** 10.1371/journal.pone.0154603

**Published:** 2016-05-03

**Authors:** Kazeem Dauda Adeyemi, Rafiat Morolayo Shittu, Azad Behnan Sabow, Mahdi Ebrahimi, Awis Qurni Sazili

**Affiliations:** 1 Department of Animal Science, Faculty of Agriculture, Universiti Putra Malaysia, 43400 UPM Serdang, Selangor, Malaysia; 2 Halal Products Research Institute, Universiti Putra Malaysia, 43400 UPM Serdang, Selangor, Malaysia; 3 Laboratory of Animal Production, Institute of Tropical Agriculture, Universiti Putra Malaysia, 43400 UPM Serdang, Selangor, Malaysia; 4 Department of Food Technology, Faculty of Food Science and Technology, Universiti Putra Malaysia, 43400 UPM Serdang, Selangor, Malaysia; 5 Department of Veterinary Preclinical Sciences, Faculty of Veterinary Medicine, Universiti Putra Malaysia, 43400 UPM Serdang, Selangor, Malaysia; 6 Department of Animal Production, University of Ilorin, PMB 1515, Ilorin, Nigeria; 7 Department of Animal Resource, University of Salahaddin, Erbil, Kurdistan Region, Iraq; CSIRO, AUSTRALIA

## Abstract

This study appraised the effects of dietary blend of 80% canola oil and 20% palm oil and *postmortem* ageing on oxidative stability, fatty acids and quality attributes of *gluteus medius* (GM) muscle in goats. Twenty-four Boer bucks were randomly allotted to diet supplemented with 0, 4 and 8% oil blend, fed for 100 days and slaughtered, and the GM muscle was subjected to a 7 d chill storage (4±1°C). Diet had no effect (*P*> 0.05) on the colour, drip loss, thiobarbituric acid-reactive substances (TBARS) value, free thiol, carbonyl, myoglobin and metmyoglobin contents, metmyoglobin reducing activity (MRA), antioxidant enzyme activities and abundance of myosin heavy chain (MHC) and actin in the GM muscle in goats. The meat from goats fed 4 and 8% oil blend had higher (*P<* 0.05) concentration of α and γ-tocopherol and abundance of troponin T compared with that from the control goats. The GM muscle from the oil-supplemented goats had lower (*P*< 0.05) concentration of C16:0 and greater (*P*< 0.05) concentration of C18:1n-9, C18:3n-3 and C20:5n-3 compared with that from the control goats. Nonetheless, diet did not affect (*P*< 0.05) the total fatty acid in the GM muscle in goats. Regardless of the diet, the free thiol and myoglobin contents, concentration of tocopherol and total carotenoids, MHC and MRA in the GM muscle decreased (*P*< 0.05) while carbonyl content, TBARS, drip loss and metmyoglobin content increased over storage. Dietary blend of 80% canola oil and 20% palm oil beneficially altered tissue lipids without hampering the oxidative stability of chevon.

## Introduction

The increase in the consumers’ awareness of the relationship between the consumption of ruminant meat and the incidence of chronic diseases has created the impetus to develop healthier ruminant meat, which would confer potential health benefits to consumers whilst enhancing consumers’ confidence in ruminant meat [1, 2]. A case in point was the recent report of the World Health Organization, which classified the consumption of red meat as “probably carcinogenic to humans” [3].

The incorporation of unsaturated fats in the diet of ruminants is an effective strategy for modifying the fatty acid composition of ruminant meat in response to the demands of the consumers [2, 4]. However, this practice could have negative impact on the oxidative stability and physicochemical properties of ruminant meat [2, 4]. In addition, *postmortem* alterations in muscle and its conversion to meat during ageing could create conditions in which the balance between antioxidant and pro-oxidant capacity favours oxidative damage [5, 6]. Oxidative deterioration of meat lipids and proteins could imperil the nutritional and sensory qualities and the shelf life of meat, and their potential perils to humans is of current interest [4, 7, 8]. Thus, attenuating oxidative deteriorations to maintain meat quality and safety is essential.

Little attention has been paid to the role of dietary fat on protein oxidation as opposed to lipid oxidation in ruminant meat. In addition, there is dearth of information on the response of myoglobin and myofibrillar proteins to dietary lipids in ruminant. The antioxidant status of muscle is the major factor influencing oxidative spoilage in muscle foods [6–8]. The potency of synthetic antioxidants in curbing *postmortem* lipid oxidation has been documented [9, 10]. Nonetheless, the potential of synthetic antioxidants causing toxicological effects [11, 12] has stimulated interest in the utilization of natural antioxidants [9, 13, 14]. In addition, synthetic antioxidant are very scarce and expensive particularly in the developing countries [13, 14]. Canola oil can be utilized in animal nutrition to alter that fatty acid profile of animal products [2, 15]. In addition, palm oil [16] and canola oil [17] are rich sources of natural antioxidants. Thus, it was hypothesized that dietary supplementation of canola oil and palm oil blend would enhance the beneficial lipids and oxidative stability of myoglobin, lipids and myofibrillar proteins in goat meat. The objective of this study was to investigate the influence of blend of 80% canola oil and 20% palm oil and *postmortem* refrigerated storage on fatty acid composition, antioxidant status, oxidative stability of myoglobin, lipid and myofibrillar proteins, physicochemical properties and metmyoglobin reducing activity in *gluteus medius* muscle in goats.

## Materials and Methods

### Animal welfare

This study was conducted following the guidelines of the research policy of the Universiti Putra Malaysia on Animal welfare and ethics. The experimental protocol was approved by the Universiti Putra Malaysia Animal use and care committee. The care of the experimental goats was in accordance to Malaysian standards.

### Animals, husbandry conditions and diets

The goats were obtained and reared at Ar-Raudhah Biotech Farm, Pty Ltd. Kuang, Selangor, Malaysia following the approval by the farm management. Twenty-four Boer crossbred bucks (4–5 months old, average initial live-weight of 20.54±0.475 kg) were drenched against parasite and randomly allotted to diets containing 0, 4 and 8% oil blend on a DM basis and fed daily for 100 d following a two-week period of adaptation. Each goat was individually housed in a wooden slated floor pen equipped with feeding and drinking facilities. Dietary treatments were formulated to meet the nutritional requirements of growing goats following the recommendation of NRC [18]. Each diet consisted of 50% concentrate mixture and 50% forage (oil palm frond) on a DM basis [19]. The oil blend replaced the corn grain in the concentrate mixture and other components of the concentrate mixture were adjusted [19] to make the diets isocaloric and isonitrogenous (Table 1). The oil-based diets were prepared by manually incorporating the oil blend into the ground concentrate followed by a thorough mixing. The diets were prepared fresh twice a day and no antioxidant was added. The diets were offered as complete ration mix (forage and concentrate) in two equal meals at 0830 and 1430 hours. All goats had *ad libitum* access to water. Feed samples (300 g) were collected weekly and stored at -20°C until analysis. Feed samples were dried at 60°C for 48 h to determine the DM content, ground to pass a through a 1 mm screen and analysed for ash, ether extract and crude protein according to the protocol of AOAC [20]. The neutral detergent fibre and acid detergent fibre were analysed by the protocol of Van Soest et al. [21].

**Table 1:** Chemical and fatty acid composition and antioxidant content of dietary treatments.

	Levels of oil blend (%)		
Chemical composition, % DM	0	4	8
Dry matter	67.70	67.90	68.07
Crude Protein	14.27	14.37	14.39
Ether extract	2.30	6.35	11.11
Organic matter	93.16	93.42	93.55
Nitrogen free extract	16.56	13.97	12.45
ADF	35.04	33.28	32.52
NDF	63.52	62.67	62.06
Metabolizable energy, MJ/Kg DM^1^	11.59	11.61	11.62
Ca	1.02	1.05	1.04
P	0.52	0.54	0.54
*Fatty acid (g/kg DM)*			
C12:0	0.01	0.03	0.04
C14:0	0.53	0.51	0.51
C16:0	2.79	5.98	7.78
C16:1	0.08	0.11	0.15
C18:0	0.56	1.12	1.43
C18:1n-9	3.82	14.87	26.32
C18:2ω-6	7.05	11.87	12.06
C18:3ω-3	1.06	2.61	4.13
n6/n3	6.65	4.55	2.92
Total FA	15.83	37.09	52.27
*Antioxidants (mg/kg)*			
Total carotenoid	14.81	16.71	19.86
α-tocopherol	101.12	112.47	123.21
γ-tocopherol	10.22	34.55	49.17
δ-tocopherol	1.21	3.45	5.93

^1^Calculated

### Slaughtering procedure and muscle sampling

The goats were fasted overnight with *ad libitum* access to water and slaughtered according to the halal procedure as outlined in MS1500:2009 [22]. After carcass dressing on day 0, 45 g of *gluteus medius* (GM) muscle, was dissected from the outer surface of the left pelvis, trimmed free of external fat and epimyseal connective tissue and divided into three equal parts. The remaining GM samples were left on the carcasses intact and removed after 1, 4 and 7 d of storage. The first part (15 g) was pulverized in liquid nitrogen to produce a homogenous powder and assigned for the determination of fatty acids, myoglobin, metmyoglobin reducing activity (MRA), antioxidants, lipid oxidation, protein oxidation, SDS-PAGE and metmyoglobin content. The second part (15 g) was vacuum packaged and stored in a chiller at 4±1°C for the determination of drip loss. The third part (15 g) was assigned for the determination of colour on d 0. Same measurements were conducted on samples taken at d 1, 4 and 7 *postmortem* from the muscle remaining on the carcasses.

### Determination muscle glycogen and pH

The glycogen content was determined using EnzyChrom^TM^ Glycogen Assay kit (Cat# E2GN-100, BioAssays, USA) following the procedure of the manufacturer. The pH of muscles was determined following the method of AMSA [23] using a portable pH meter (Mettler Toledo, AG 8603, Switzerland). The pH meter was calibrated with a pH 4.0 buffer and then with a pH 7.0 buffer prior to use. Each pulverized sample (0.5 g) was homogenized with 10 mL ice-cold water in the presence of 5 mM sodium iodoacetate to prevent further glycolysis. The pH of the resultant homogenate was measured at 20±1°C.

### Determination of Drip loss

Drip loss was measured as described by Sabow et al. [24]. The fresh meat samples from the GM muscle on d 0 were weighed and recorded as initial weight (W1). The weighed samples were placed into polyethylene plastic bags, labelled, vacuum packaged and stored at 4°C. After 1, 4 and 7 d *postmortem*, the samples were removed from the bags, gently blotted dried, weighed and recorded as W_2._ Drip loss was calculated and expressed as the percentage of the difference between the initial and the final weight of sample after storage divide by the initial weight of sample as shown in the equation below:

$$Drip\;loss\;(\%)=\left[\frac{\left(W1-W2\right)}{W1}\right]\times 100$$

### Determination of colour

Meat colour coordinates were determined using a Colour Flex spectrophotometer (Hunter Lab Reston, VA, USA) based on the International Commission on Illumination (CIE) Lab-values (also known as lightness (L*), redness (a*) and yellowness (b*) with D_65_ illuminant and 10° standard observer, tristimulus values (X,Y,Z) and reflectance at specific wavelength (400–700) nm [23]. The device was calibrated against black and white reference tiles prior to use. The samples were bloomed for 30 min and placed at the base of the colour flex cup. For each sample, three readings for each of L*, a* and b* values were recorded and averaged.

### Determination of myoglobin, metmyoglobin content and metmyoglobin reducing activity

The extraction and quantification of myoglobin followed the method of Warriss [25]. The metmyoglobin content was determined as described by Krzywicki [26]. The extraction of myoglobin reductase and the determination of metmyoglobin reducing activity (MRA) in pulverized meat samples followed the procedure of Mikkelsen et al. [27].

### Fatty acid analysis

Total lipids in feed and meat samples were extracted in chloroform:methanol (2:1, v/v) mixture according to the method of Folch et al. [28] modified by Rajion et al. [29]. The extracted lipids were transmethylated to their fatty acid methyl esters (FAME) using 2 mL 14% BF_3_ and 2 mL 0.66 N KOH in methanol according to the method of AOAC [20]. The FAME was separated in a gas liquid chromatograph (Agilent 7890A, Agilent Technologies, Inc., anta lara, CA) equipped with a flame ionization detector using a 100 m x 0.25mm ID (0.20 μm film thickness) Supelco SP-2560 capillary column. Helium was the carrier gas and the split ratio after the FAME injection was 10:1. The temperature of the injector and detector were programmed at 250°C and 300°C, respectively. The column temperature was set at 100°C, held for 2 min and warmed to 170°C at 0°C/min, held for 2 min, warmed to 230°C at 5°C/min and then held for 20 min. The fatty acid composition of sample was determined by comparing the relative retention times of FAME peaks from samples with those from standards.

### Determination of lipid oxidation

Lipid oxidation was measured as 2-thiobarbituric acid reactive substances (TBARS) using QuantiChrom^TM^ TBARS Assay Kit (DTBA-100, BioAssay Systems, USA) following the description of the manufacturer.

### Antioxidant Enzyme activity

Glutathione peroxidase (GPX) activity was measured with the aid of EnzyChromTM glutathione Peroxidase Assay Kit EGPX-100, (BioAssay Systems, USA) following the manufacturer’s protocol. Superoxide dismutase (SOD) activity was measured using Cayman SOD Assay kit 706002, (Cayman chemical) following the manufacturer’s protocol. Catalase (CAT) activity was measured using Cayman Catalase Assay Kit 707002, (Cayman chemical) following the manufacturer’s procedure.

### Determination of total carotenoid

The carotenoid contents in feed and meat samples were extracted and quantified following the method described by Okonkwo [30]. Two gram of each sample was homogenized with 10 mL acetone. The contents were stirred for 30 min and two 5 mL aliquot of acetone was used to rinse the flask and re-extract the residue. The extracts were pooled and 1 mL of deionized water was added. The mixture was transferred into 5 mL n-hexane and centrifuged at 3000 g for 10 min. The absorbance of the hexane layer was read at 450 nm using a spectrophotometer (Secomam, Domont, France). The total carotenoid contents was estimated by the following formula:

$$Concentration\;\left(\frac{\mu }{g}\right)=\frac{\left(A\times V\times 10^{4}\right)}{\left(A1\%/1cm\times W\right)}$$

Where A = absorbance

V = Volume of n-hexane (mL)

W = Sample weight

A1%/1cm = 2592 (absorption coefficient of carotene)

### Determination of tocopherol

The extraction of tocopherol from feed and tissue samples followed the method of Kamal-Eldin et al. [31]. The tocopherol contents were quantified using Agilent 1200 series HPLC as described by Pegg and Amarowicz [32]. The column used was C_30_ YMC^TM^ carotenoid (250 mm x 4.6 mm. i.d, 5 μm) (YMC, USA). An isocratic mobile phase made up of 99% n-hexane and 1% Isopropanol was used. The flow rate was 0.5 mL/min and the injection volume was 20 μL. UV detection was monitored at 295 nm. The isomers of tocopherol were quantified by comparing the peak area of samples with those of tocopherol standards in the HPLC controller software.

### Determination of carbonyl and free thiol contents

Protein thiol content was quantified according to the Elman’s method using 2,2-dithiobis (5-nitropyridine) DTNP [33]. The results were expressed as nmol/mg protein. The carbonyl content in muscles was determined using Cayman protein carbonyl colorimetric assay kit (10005020) following the manufacturer’s procedure. Carbonyl content was expressed as nmol/mg protein.

### Sodium dodecyl sulphate polyacrylamide gel electrophoresis (SDS-PAGE) and western blotting

The myofibrillar proteins were extracted with an extraction buffer containing 3 mM MgCl_2_, 25 mM KCl, 4 mM EDTA and 150 mM NaCl at a pH of 6.5 following the method of Morzel et al. [34]. The protein concentration of the samples was determined following the Bradford method [35] using Protein Assay Kit II 500–0002 (Bio-Rad, Hercules, CA, USA). Bovine serum albumen (BSA) was used to prepare the protein standards.

Myofibrils were incubated at 90°C for 10 min in a buffer containing 2.3% (w/v) SDS, 62.5 mM Tris–HCl (pH 6.8), 5% (v/v) mercaptoethanol, 0.05% (w/v) bromophenol blue and 30% (v:v) glycerol. One dimensional SDS-PAGE was performed according to the method of Laemmli [36] using polyacrylamide gels of 8 cm × 5.5 cm (length × width) and 0.8 mm thickness. The resolving gels were over-layered with 4% stacking gel solution. Samples (30 μg protein each) were separated in running buffer (0.025 mol/L Tris base, 0.192 mol/L glycine, 0.1 SDS, pH 8.3) using a mini PROTEAN® Tetra system (Bio-Rad) set at a constant voltage of 120 V and a current of 0.4 A for 90 min. Coomassie blue stain (0.05% Coomassie blue, 5.0% acetic acid, 15% methanol) was used to stain the gels for 60 min. Thereafter, the gels were destained with destaining solution (10% acetic acid and 30% methanol) for 45 min to remove excessive background. The bands of myofibrillar proteins were visualized using a GS-800 Calibrated Imaging Densitometer (Bio-Rad, USA).

The electrophoresed proteins were transferred from the gel onto polyvinylidene difluoride (PVDF) membranes using Trans-Blot® SD semi-dry transfer system cell (Bio-Rad, USA). Myosin heavy chain was transferred at constant amperage of 250 mA per gel, voltage limit of 25 V for 135 min while actin and troponin-T were transferred at the same amperage and voltage for 45 min. The membranes were immersed in ponceau staining solution (0.5% ponceau S and 5% trichloroacetic acid) for 5 min to visualize the proteins of interest and to verify the electrophoretic transfer. The membranes were washed with deionized water thrice and later washed with TBST buffer (100 mM Tris-HCl, 150 mM NaCl and 0.05% Tween 20) once. The membranes were blocked with blocking buffer (5% BSA in TBST buffer) for 3 h at room temperature (26°C) with constant shaking (Multi Shaker-FMS3-FINEPCR, Korea). Thereafter, the membranes were incubated overnight with 1: 500 dilution of primary antibody. The primary antibody used for myosin heavy chain (fast), myosin heavy chain (slow), actin and troponin-T were Monoclonal Anti-Myosin (Skeletal, Fast, produced in mouse; Cat # M4276, Sigma- Aldrich, USA), Monoclonal Anti-Myosin (Skeletal, Slow, produced in mouse; Cat # M842, Sigma- Aldrich, USA), monoclonal Anti-actin (produced in rabbit; Cat # A2066 227, Sigma- Aldrich, USA) and monoclonal anti-troponin T (produced in mouse; Cat # T6277, Sigma- Aldrich, USA) respectively. Subsequently, the membranes were washed three times in TBST buffer (5 min incubation at 26°C) with constant shaking). The membranes were further incubated at 26°C in 1:10000 dilution of secondary antibody [anti- mouse IgG (whole molecule)-peroxidase, antibody developed in rabbit; Cat # A9044, Sigma- Aldrich, USA] in 3% BSA in TBS-T buffer for 90 min. The membranes were washed thrice with TBST buffer. The blocked membranes were detected using a DAB substrate kit Code: E733, DAB SUBSTRATE SYSTEM (aMReSCO®, Ohio). The band intensity of myofibrillar proteins was measured by Quantity one® software on GS-800 Calibrated Imaging Densitometer (Bio-Rad, USA).

### Statistical analysis

The experiment followed a completely randomized design model. Data obtained were analysed using the PROC MIXED procedure of SAS [37] in which diet, *postmortem* ageing and interaction between diet and *postmortem* ageing were fitted as fixed effects in a repeated measure analysis of variance. Means were separated by Tukey HSD test at significant level of *P*< 0.05.

## Results and Discussion

### Muscle pH, glycogen and drip loss

Dietary oil blend did not affect (*P*> 0.05) the glycogen content and pH of GM muscle throughout the *postmortem* storage (Table 2). This observation could be due to the comparable available energy from the dietary treatments and the homogenous management and slaughter conditions employed during the trial [19]. Dietary energy and *antemortem* stress affect the concentration of muscle glycogen at the time of slaughter [38, 39].

**Table 2:** Mean physicochemical properties of *gluteus medius* muscle in goats as influenced by dietary oil blend and *postmortem* ageing.

	Level of oil blend (%)				Storage days					*P value*		
Parameter	0	4	8	SEM	0	1	4	7	SEM	diet	days	diet x days
Glycogen mg/g	1.07	1.07	1.07	0.12	1.30^a^	0.58^b^	0.56^b^	0.57^b^	0.01	0.312	0.031	0.213
pH	5.61	5.63	5.64	0.45	6.34^a^	5.60^b^	5.60^b^	5.59^b^	0.19	0.102	0.002	0.221
Drip loss (%)	6.71	6.67	6.60	0.44	-	5.89^c^	6.91^b^	7.60^b^	0.11	0.234	0.001	0.237
L*	31.49	30.41	33.09	1.27	34.04^b^	34.98^b^	37.22^a^	38.02^a^	1.59	0.138	0.034	0.106
a*	12.04	12.38	12.79	0.90	11. 21^a^	12.02^a^	10.23^b^	9.45^c^	0.72	0.041	0.002	0.219
b*	12.77	13.75	12.60	0.94	13.14	12.98	12.47	12.33	0.31	0.512	0.128	0.106
Myoglobin (mg/g)	2.80	2.81	2.84	0.07	2.89^a^	2.80^a^	2.71^b^	2.60^b^	0.08	0.111	0.041	0.223
Metmyoglobin (%)	6.69	6.55	6.56	0.08	2.91^d^	7.39^c^	12.35^b^	18.99^a^	3.12	0.222	< .0001	0.441
MRA (nmol min^-1^g^-1^)	212.4	210.2	212.7	14.0	212.7^a^	200.5^b^	190.2^c^	181.9^d^	10.22	0.516	0.021	0.354

a, b, c means having different superscripts along the same row for each factor are significantly different.

L* = lightness. a* = redness. b* = yellowness. MRA = metmyoglobin reducing activity. SEM = standard error of mean

Regardless of the diet, the muscle glycogen content and pH observed on d 0 were greater (*P*< 0.05) than those observed on other storage days. The finding could be due to *postmortem* glycolysis. After slaughter, the dwindling oxygen supply shifts muscle metabolism from aerobic to anaerobic, which breaks down available muscle glycogen via the glycolytic pathway to form lactic acid that lowers the muscle pH [39, 40]. The stability of the muscle pH and glycogen on d 1, 4 and 7 *postmortem* indicates that *postmortem* glycolysis was completed at 24 h *postmortem*. The breakdown of glycogen to lactic acid continues until a pH is reached when the enzymes effecting the conversion of glycogen to lactic acid become inactivated [38–40].

Diet had no effect (*P*< 0.05) on the drip loss of GM muscle in goats (Table 2). The current finding agrees with that of Mir et al. [41] who observed that the drip loss in beef from steers fed sunflower oil with or without vitamin E was similar to that from the control steers. Drip loss increased (*P*< 0.05) over storage. This observation could be due to the decrease in the available space (steric effects) for water to reside in the muscle due to the formation of cross-bridges between the thick and thin myofibrillar filaments during rigor development [39, 42]. The current observation is consistent with that of Sabow et al. [24].

### Colour, myoglobin, metmyoglobin and metmyoglobin reducing activity

The colour coordinates, myoglobin and metmyoglobin contents and metmyoglobin reducing activity of GM muscle in goats are shown in Table 2. Colour is an important meat quality attribute because it is the first tool used by consumers for the identification and selection of meat [38, 39]. Diet had no effect (*P*> 0.05) on the redness, yellowness and lightness of GM muscle in goats. This finding could be attributed to the similar muscle myoglobin and metmyoglobin contents and metmyoglobin reducing activity in the meat of goats fed different diets. This observation is in agreement with that of Haak et al. [7] who observed that dietary supplementation of oxidized linseed oil with or without antioxidant had no effect on the colour coordinates of pork. In contrast, Jensen et al. [43] observed that dietary rapeseed oil enhanced pork redness.

The meat redness decreased (*P*< 0.05) while the meat lightness increased (*P*< 0.05) over storage. This finding could be due to the decrease in myoglobin concentration and metmyoglobin reducing activity (MRA) and the increase in metmyoglobin content over storage. Oxidation of muscle myoglobin to metmyoglobin can lead to discoloration of red meat [39, 44]. Previous studies have demonstrated that meat lightness increased while meat redness decreased during *postmortem* ageing of mutton [44], chevon [24] and beef [45].

Myoglobin is the major determinant of meat colour [39]. Diet did not affect the myoglobin concentration in GM muscle in goats. The decrease in the concentration of myoglobin could be responsible for the increase in the metmyoglobin content over storage. Similarly, the metmyoglobin content in beef patties subjected to a 10 d [9] refrigerated storage increased over storage. The MRA decreased (P< 0.05) over storage. This observation is in agreement with that of Madhavi and Carpenter [46] who observed a reduction in the MRA of beef during chill storage. In contrast, there was a significant increase in the MRA of beef patties during a 10 d chill storage [9]. In addition, the MRA of ovine *longissimus* [44] and beef patties [47] was stable during a 10 d and 9 d *postmortem* ageing respectively.

### Fatty acid composition

The fatty acid (FA) composition of g*luteus medius* muscle in goats is shown in Table 3. Regardless of the diet, the three most abundant FA were C16:0, C18:0 and C18:1n-9. Similar trends were reported for chevon [2] and mutton [48]. The meat from goats fed 4 and 8% oil blend had lower (*P*< 0.05) concentration of C16:0 and C16:1n-7 compared with that from the control goats. This observation could be due to the displacement or dilution effects of other fatty acids, the decrease in the activity of lipogenic enzymes responsible for the synthesis of medium chain FA or the preferential incorporation of long chain FA from diet and/or adipose tissues [15, 49, 50]. Despite the clear differences between tissue FA deposition and milk FA secretion, the basic characteristics of ruminant lipid metabolism could be invoked in order to utilize information obtained in dairy cows for interpreting the meat composition in lambs, cattle and goats [49]. This finding is consistent with that of Nicole et al. [15] who found that the supplementation of 10.4% canola oil or flaxseed oil reduced the concentration of C16:0 and C16:1n-7 in cow milk. Similarly, Otto et al. [50] observed that the supplementation of crude degummed canola oil reduced the concentration of C16:0 in cow milk.

**Table 3:** Mean fatty acid composition (mg/ 100 g meat) of *gluteus medius* muscle in goats as influenced by dietary oil blend and *postmortem* ageing.

Fatty acids	Levels of oil blend (%)				Storage time (days)				*P value*		
	0	4	8	SEM	0	4	7	SEM	Diet	storage	dietxstorage
C14:0	88.96	89.75	80.82	7.29	89.36^a^	92.47^b^	97.33^c^	6.90	0.736	0.031	0.312
C16:0	635.66^a^	600.95^b^	580.55^c^	14.18	617.21^a^	640.22^b^	667.11^c^	12.00	0.022	0.003	0.583
C16:1n-7	89.28^a^	70.31^b^	67.30^c^	6.21	84.22	80.23	78.19	5.12	0.017	0.082	0.783
C18:0	573.78	565.78	495.97	20.11	560.21	578.12	590.20	15.87	0.375	0.213	0.091
C18:1n-9	908.19^b^	974.93^a^	995.81^a^	31.34	950.41^a^	908.22^b^	900.34^c^	27.45	0.647	0.040	0.777
C18:1*trans*-11	48.64	56.57	41.89	3.43	50.12	52.31	51.00	4.44	0.078	0.134	0.778
*cis-*9 *trans-*11 CLA	30.72	38.23	33.49	2.16	35.23	38.11	36.16	2.19	0.526	0.219	0.605
*trans-*10 *cis-*12 CLA	37.76	41.71	41.88	4.17	39.21	40.11	39.54	3.00	0.113	0.214	0.203
C18:2n-6	399.53	397.25	397.93	14.11	380.21^a^	350.11^b^	341.97^c^	12.10	0.167	0.001	0.479
C18:3n-3	59.12^a^	68.23^b^	87.32^c^	2.00	38.21^a^	32.15^b^	28.22^c^	1.98	0.030	0.021	0.239
C20:4n-6	199.13	153.81	156.71	14.22	200.10^a^	189.21^b^	170.45^c^	10.23	0.109	0.003	0.196
C20:5n-3	48.56^a^	61.28^b^	92.40^c^	5.45	70.15^a^	61.34^b^	53.32^c^	6.12	0.038	0.028	0.144
C22:5n-3	30.64	57.07	76.62	6.16	56.15^a^	41.23^b^	34.22^c^	7.80	0.072	0.045	0.347
C22:6n-3	50.16	60.66	68.57	6.11	70.22^a^	65.23^b^	60.09^c^	5.28	0.061	0.011	0.104
Total FA	3200.13	3236.54	3217.26	25.61	3060.81	3169.06	3113.92	23.23	0.218	0.567	0.284
Fatty acid sum and ratios											
∑SFA	1298.35^a^	1256.48^b^	1157.34^b^	30.22	1266.78	1310.81	1354.64	32.11	0.041	0.001	0.327
∑MUFA	1046.11	1101.81	1105.00	22.16	1084.75	1040.76	1029.53	21.45	0.586	0.056	0.682
∑PUFA	855.62^b^	878.24^b^	954.92^a^	21.07	889.48^a^	817.49^b^	763.97^c^	20.00	0.001	0.001	0.174
∑ω-3	188.48^a^	247.24^b^	324.91^c^	10.13	234.73^a^	199.95^b^	175.85^c^	10.13	0.028	0.001	0.778
∑ω-6	598.66	551.06	554.64	17.12	580.31^a^	539.32^b^	512.42^c^	15.78	0.004	0.011	0.788
ω-6:ω-3	3.18^a^	2.23^b^	1.71^c^	0.26	2.48	2.69	2.91	0.17	0.015	0.217	0.725
UFA:SFA	1.46	1.57	1.78	0.08	1.56	1.42	1.32	0.03	0.022	0.116	0.299
PUFA:SFA	0.65^a^	0.69^b^	0.83^c^	0.05	0.70	0.62	0.79	0.04	0.038	0.291	0.243

a, b, c means having different superscripts along the same row for each factor are significantly different.

SEM = standard error of mean

The concentration of C18:1n-9 was greater (*P*< 0.05) in the meat from the oil-fed goats compared with that from the control goats. This observation could be due to the increase in the intake of C18:1n-9 as the level of the oil blend increased in diet [19]. In addition, the increase in C18:1n9 could be due to the delta-9 desaturation of 18:0 in tissues [15, 49, 50]. This observation is in agreement with the report of a companion *in vitro* study [51], which showed that the concentration of C18:1n-9 after 24 h incubation increased in response to incremental level of oil blend in the substrate. Similarly, dietary canola oil [15] and crude degummed canola oil [50] increased the concentration of C18:1n-9 in bovine milk.

The concentrations of 14:0, C18:0, C18:1*trans*11, C18:2n-6, CLA cis-9 *trans*-11 and CLA cis-12 *trans*-10 and C20:4n-6, C22:5n-3, C22:6n-3 and total FA in the GM muscle were not influenced (*P>* 0.05) by diet. The concentration of C18:3n-3 and C20:5n-3 was greater (*P<* 0.05) in the meat from the oil-supplemented goats compared with that from the control goats. The increase in the concentration of C18:3n-3 could be due to the increase in the dietary intake of C18:3n-3 [19] in response to the incremental level of the oil blend in the diet. The increase in the concentration of C20:5n-3 could be due to the elongation of C18:3n-3. Similarly, dietary C18:3n-3 enhanced the concentration of C18:3n-3 and its long chain derivatives in lambs [49]. The PUFA/SFA increased (*P<* 0.05) while the n6/n3 decreased (*P<* 0.05) as the level of the oil blend increased in diet. Similarly, Karami et al. [2] observed that goats fed canola oil had greater concentration of C18:3n-3 and lower n6/n3 in *longissimus lumborum* muscle in goats compared with those fed palm oil.

*Postmortem* ageing influenced (*P*< 0.05) the FA composition of GM muscle in goats. The concentration of C18:1n-9, n-3 and n-6 FA and total polyunsaturated fatty acids (PUFA) decreased (*P*< 0.05) while the concentration of C14:0 and C16:0 increased (*P*< 0.05) over storage. This observation could be attributed to the decrease in the concentration of tocopherols and total carotenoids (Table 4) and the increase in lipid oxidation over storage. Similarly, Muíño et al. [4] observed a decrease in the concentration of n-3 and n-6 PUFA in mutton subjected to chill storage for 12 d.

**Table 4:** Mean antioxidant contents, lipid and protein oxidation in *gluteus medius* muscle from goats as influenced by dietary oil blend and *postmortem* ageing.

	Level of oil blend^ ^(%)				Storage days				*P value*		
Parameter	0	4	8	SEM	0	4	7	SEM	diet	storage	diet x storage
α-tocopherol (mg/ kg)	2.46^b^	3.38^a^	4.02^a^	0.26	3.24^a^	2.92^b^	2.90^c^	0.30	0.001	0.023	0.101
γ-tocopherol (mg/kg)	0.47^b^	0.77^a^	0.98^a^	0.08	0.93^a^	0.85^b^	0.80^b^	0.06	0.001	0.008	0.243
δ-tocopherol (mg/kg)	0.06	0.08	0.08	0.01	0.07^a^	0.05^b^	0.03^c^	0.01	0.196	0.010	0.311
Carotenoid (mg/kg)	0.24	0.26	0.29	0.07	0.25^a^	0.20^b^	0.18^b^	0.08	0.142	0.014	0.099
TBARS (mg MDA/kg)	0.21	0.23	0.20	0.02	0.15^a^	0.19^b^	0.38^c^	0.03	0.418	<0.001	0.173
Free thiol (nmol/mg protein)	54.73	54.51	54.62	0.89	54.62^a^	43.06^b^	40.34^c^	1.00	0.691	0.037	0.534
Carbonyl (nmol/mg protein)	3.16	3.09	3.10	0.04	1.23^c^	3.45^b^	5.87^a^	0.67	0.584	< .001	0.125
Myosin heavy chain fast (density/mm^2^)	41.45	43.76	45.50	3.22	46.78	42.33	37.38	1.92	0.502	0.023	0.564
Myosin heavy chain slow (density/mm^2^)	70.10	74.14	76.13	2.13	76.12^a^	71.03^b^	64.20^c^	3.00	0.234	0.016	0.213
Actin (density/mm^2^)	16.27	16.34	16.61	0.21	16.67	16.33	16.15	0.67	0.127	0.124	0.145
Troponin T (density/mm^2^)	13.22^b^	15.26^a^	15.30^a^	0.76	15.99^a^	14.00^b^	12.82^c^	0.17	0.012	0.001	0.219

a, b, c means having different superscripts along the same row for each factor are significantly different. SEM = standard error of mean

### Antioxidant status, lipid and protein oxidation

The antioxidant status and oxidative stability of chevon in response to dietary oil blend and *postmortem* ageing are presented in Table 4. Diet did not affect (*P*> 0.05) the catalase, superoxide dismutase and glutathione peroxidase activities in the GM muscle in goats. This observation is an indication that the oil blend did not instigate oxidative stress in the goats. It has been demonstrated that animals exposed to dietary oxidative stress respond with compensatory induction of antioxidant enzymes [52, 53]. The current observation contradicts that of Renerre et al. [53] who observed that dietary rapeseed oil increased the antioxidant enzyme activities in turkey meat. *Postmortem* ageing did not affect (*P>* 0.05) the antioxidant enzyme activities in the GM muscle in goats. Similarly, the GPX and CAT activities in different bovine muscles on d 1 were similar to those observed on d 8 *postmortem* [45]. In contrast, the antioxidant enzyme activities in the breast and thigh muscles of Korean chickens decreased over a 10 d *postmortem* chill storage [54].

Dietary supplementation of oil blend enhanced (*P<* 0.05) the concentration of α and γ-tocopherol in the GM muscle in goats. This observation presumably reflects the antioxidant contents of the dietary treatments. Tocopherol is a fat-soluble vitamin [43]. Thus, the increase in the dietary fat might have aided the absorption and deposition of tocopherol in the tissue of the oil-fed goats. Similarly, Soler-Velasquez et al. [55] observed that dietary canola oil increased the α-tocopherol content in pork. Diet did not affect the concentration of total carotenoids and δ-tocopherol in goat meat. Regardless of the diet, the concentration of total carotenoids and α, γ, and δ-tocopherol in GM muscle decreased (*P*< 0.05) over storage. Similar finding was observed during *postmortem* ageing of beef [56]. The interaction between diet and postmortem ageing was not significant for the concentration of total carotenoids and α, γ, and δ-tocopherol in goat meat.

Diet did not affect (*P*> 0.05) the TBARS value of GM muscle in goats. Although the meat from the oil-fed goats had greater concentration of n-3 PUFA and consequently presented greater potential for lipid oxidation, the TBARS value was similar to that from the control meat. This observation could be due to the increase in the concentration of α and γ-tocopherol in the meat from the oil-fed goats. Regardless of the dietary treatment, the TBARS value increased as storage progressed. Similarly, *postmortem* chill storage increased TBARS value in pork [7], mutton [57] and chevon [58].

Free thiol group and carbonyl content are important indicators of protein oxidation in muscle foods [7, 8]. Diet did not affect (*P*> 0.05) the free thiol and carbonyl contents of GM muscle in goats (Table 4). These observations concur with those of Lund et al. [8] who observed that dietary soybean oil had no effect on the carbonyl content and free thiol group in porcine *quadriceps femoris* subjected to a 7 d chill storage. Similarly, dietary oxidized linseed oil with or without antioxidants did not affect the carbonyl content and free thiol group in raw and cooked pork refrigerated for 8 d [7]. Irrespective of the diet, the free thiol content decreased (*P*< 0.05) while carbonyl content (*P*< 0.05) increased as chill storage progressed. Similar findings were observed during *postmortem* ageing of pork [7, 8], mutton [59] and beef [45]. There was no significant interaction between diet and *postmortem* chill storage for carbonyl and free thiol contents in goat meat.

### Myofibrillar protein profile

**Fig 1:**
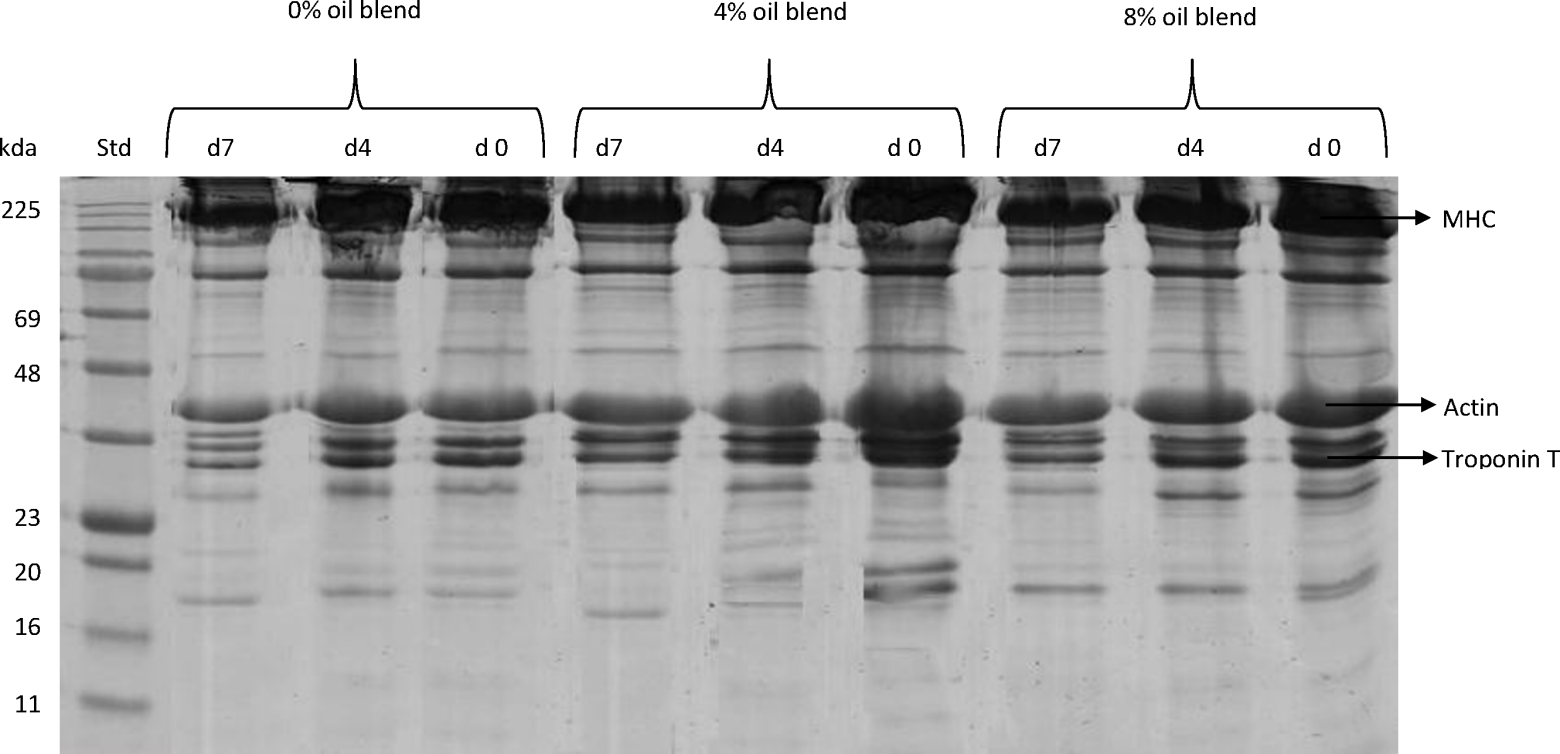
SDS-PAGE of myofibrillar proteins in gluteus medius muscle in goats. Equal amounts of protein (30 μg) of each sample was loaded and electrophoresed on a separate 12% sodium dodecyl sulfate polyacrylamide gel electrophoresis (SDS-PAGE) at a constant voltage (120 V) for 90 min. The gels were stained with Coomassie blue staining for 60 min and destained with destaining solution for 45 min. d0 = day 0, d4 = day 4, d7 = day 7. Std = standard. MHC = myosin heavy chain.

**Fig 2:**
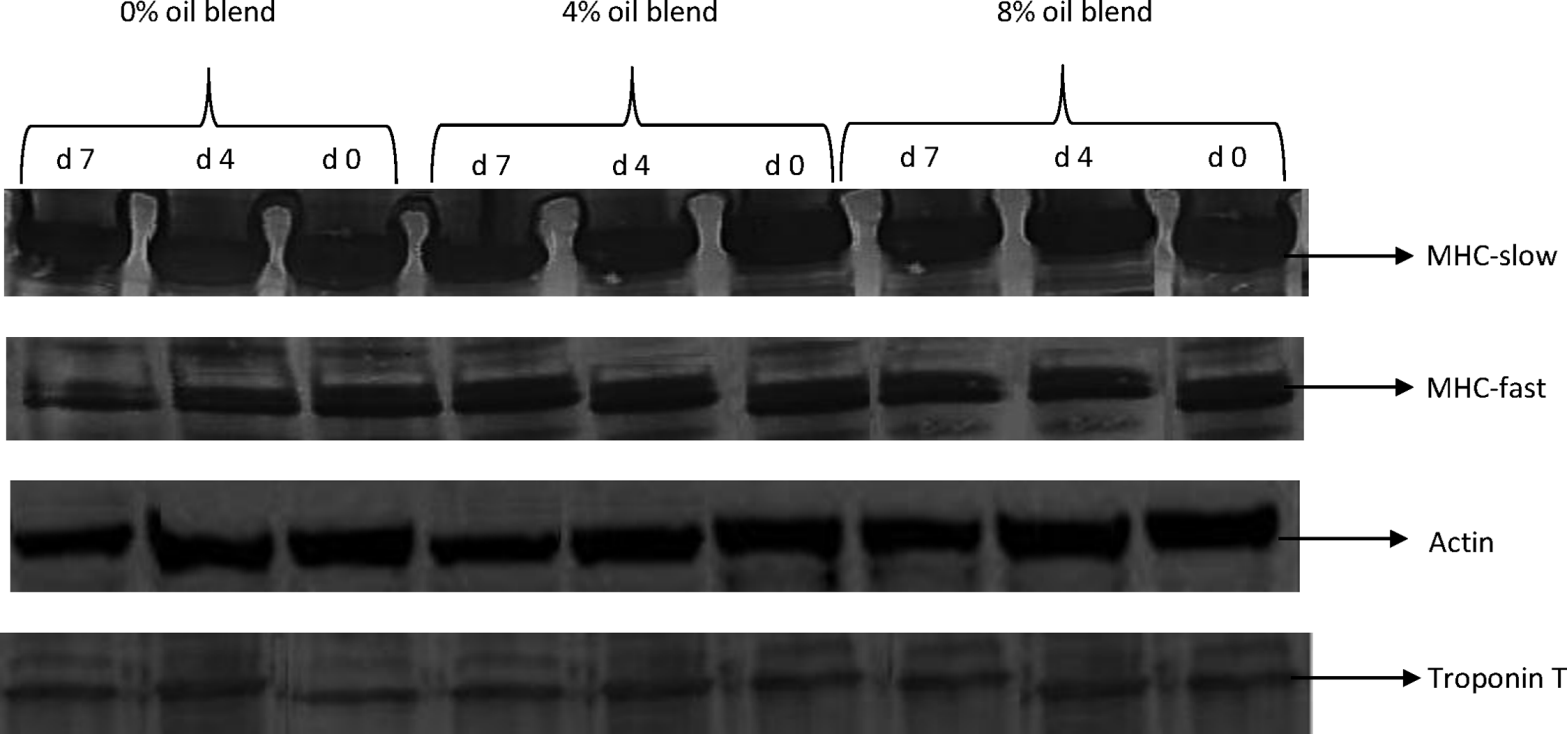
Western blot analysis of myofibrillar proteins in gluteus medius muscle in goats. d0 = day 0, d4 = day 4, d7 = day 7. MHC = myosin heavy chain.

The SDS-PAGE pattern of myofibrillar proteins in GM muscle in goats is shown in Fig 1. The reflective density of myofibrillar proteins in the GM muscle is shown in Table 4. Fig 2 presents the representative Western blots of myofibrillar proteins in GM muscle in goats. Diet did not affect (*P*> 0.05) the reflective density of myosin heavy chain fast (MHC-f), myosin heavy chain slow (MHC-s) and actin. The GM muscle from the oil-supplemented goats had greater (*P*< 0.05) reflective density of troponin T than that from the control goats. This observation could be attributed to the greater concentration α and γ-tocopherol in the GM muscle of the oil-fed goats.

Irrespective of the diet, the reflective density of MHC-f and MHC-s decreased (*P*< 0.05) over storage. Similar observations were observed during *postmortem* ageing of beef [60] and chevon [61]. In contrast, myosin heavy chain of beef *semimembranosus* was stable throughout a 28 d chill storage at 4°C [62]. The reflective density of troponin T decreased (*P*< 0.05) over storage. This observation concurs with that of Sabow et al. [61] who observed a decrease in the reflective density of troponin T of *longissimus lumborum* muscle in goats during a 14 d *postmortem* chill storage. *Postmortem* ageing was not a significant of variation affecting the reflective density of actin. This observation could be due to the masking of oxidation sites caused by the interaction of actin with myosin chain in myofibrillar suspensions [34]. Similar observation was observed during a 14 d chill storage of chevon [61].

## Conclusion

The results of this study demonstrate that dietary blend of 80% canola oil and 20% palm oil beneficially altered muscle lipids without hampering the oxidative stability of myoglobin, lipids and myofibrillar proteins and the physicochemical properties of goat meat. *Postmortem* ageing encouraged oxidative deterioration of lipids, myoglobin and myofibrillar proteins in goat meat.
